# Safety of the application of Rigidfix cross-pin system via different tibial tunnels for tibial fixation during anterior cruciate ligament reconstruction

**DOI:** 10.1186/s12891-020-03645-z

**Published:** 2020-11-11

**Authors:** Jian Wang, Hua-qiang Fan, Wenli Dai, Hong-Da Li, Yang-pan Fu, Zhenhuang Liu, Chang-ming Huang, Zhanjun Shi

**Affiliations:** 1 Department of Orthopaedic Surgery, Nanfang hospital, Southern Medical University, Guangdong, 510515 People’s Republic of China; 2 Department of Orthopaedic Surgery, Chenggong Hospital Affiliated to Xiamen University, Xiamen, 361003 Fujian Province People’s Republic of China; 3 Institute of Sports Medicine, Beijing Key Laboratory of Sports Injuries, Peking University Third Hospital, Beijing, 100191 People’s Republic of China

**Keywords:** Anterior cruciate ligament, Rigidfix, Tibia, Bone tunnel, Iatrogenic injury

## Abstract

**Background:**

We investigate the safety of the application of the Rigidfix cross-pin system via different tibial tunnels in the tibial fixation during anterior cruciate ligament (ACL) reconstruction.

**Methods:**

Five adult fresh cadaver knees were fixed with the Rigidfix cross-pins in the tibial fixation site during ACL reconstruction. Two different tibial tunnel groups were established: in group A, the tunnel external aperture was placed at the 25° angle of coronal section; in group B, the tunnel external aperture was placed at the 45° angle of coronal section. The guide was placed at the plane 0.5 mm below articular facet through the tibial tunnel, with three rotation positions set at 0°, 30°, and 60° slopes. The incidences of iatrogenic injuries at tibial plateau cartilage (TPC), medial collateral ligament (MCL), and patellar tendon in three different slope angles were calculated in groups A and B and the results were analyzed by using chi square test.

**Results:**

The iatrogenic injuries at MCL, TPC, and patellar tendon could occur after the Rigidfix cross-pin system was placed 5 mm below tibial plateau cartilage for ACL reconstruction. The incidences of TPC injury (χ^2^ = 5.662, *P* = 0.017) and MCL injury (*P* = 0.048, Fisher exact probability method) were significantly lower in group A than in group B. However, the incidence of patellar tendon injury showed no significant difference between these two groups (*χ*^2^ = 0.120, *P* = 0.729).

**Conclusions:**

When the Rigidfix cross-pin system is used for ACL reconstruction at the tibial fixation site, the external aperture of tibial tunnel should not be placed at the excessively posterosuperior site, to avoid MCL and TPC injuries.

## Background

Anterior cruciate ligament (ACL) is a key structure in maintaining the stability of knee joint. Once ACL is injured, the anterior and posterior portions of the knee joint and its partial rotation function become unstable, leading to degenerative changes of knee joint, which can induce traumatic arthritis and affect the daily life; in severe cases, the patients may even partially lose their work ability [1]. Fixation method during ACL reconstruction is a key factor that can affect the clinical outcomes [2].

The Rigidfix cross-pin system can fix the grafts at the site near the ACL physiological attachment point and thus achieve anatomic reconstruction. It can effectively reduce the “rubber band effect” and “rain-wiper effect”. Meanwhile, it can raise the chance of tendon-bone healing by achieving the 360° contact between grafted tendon and bone tunnel. Therefore, the Rigidfix cross-pin system has been widely applied in ACL reconstruction [3, 4]. However, the system can cause complications such as penetration and/or tear of the fixation when it is applied at the femoral side due to its complicated operation [5]. Many anatomic studies have been performed to standardize its application at the femoral side [6–8]. Some authors [9, 10] applied this system at the tibial side. The Rigidfix Cross-pin System applied at femoral and tibial sides, can reduce the dependence of ACL reconstruction on the graft length, which allows more flexible graft selection. However, it is still questionable that such procedures will be accompanied by iatrogenic injuries such as graft penetration or peripheral ligament injuries. Our previous research focused on anti-corrosion specimens and we proposed a “safe angle” for the operation of Rigidfix Cross-pin system in ACL reconstruction tibial fixation [11]. However, since our previous experiment used antiseptic specimens, we focused on whether the distal end of the cross pin penetrated the bone cortex and ignored the observation of internal fixation on the soft tissue damage around the knee joint. Meanwhile, in order to obtain the ideal ACL femoral reconstruction point, many scholars suggest that when drilling the tibial tunnel, the position of the tibial tunnel is shifted posteriorly, medially, and the angle of the coronal plane of the tibial tunnel is increased to 40 ~ 45°, making it easier to find the anatomical reconstruction point of the femoral tunnel through the tibial tunnel [12]. In our previous experiments, the angle between the coronal planes of the tibial tunnel was defined as 20° ~ 25° based on past techniques [13]. So, will the changes in the tibial tunnel affect the safety of the Rigidfix Cross-pin system in ACL reconstruction of the tibial side? In order to make up for the shortcomings of the previous experiment, we assume that the application of the Rigidfix Cross-pin system at the tibial side can cause complications of cross-pins and possible trauma to ligament tendons, nerves, blood vessels, etc. around the knee joint. At the same time, the incidence of iatrogenic injury through the tunneling of the cross-pins through different angles of the tibial tunnel will also be different. Therefore, we used fresh specimens for this experiment. Based on the different angles of the tibial tunnel, it is divided into two groups of 45° and 25°. In order to provide a reference for the application of Rigidfix in the tibia side, we observed the incidence of iatrogenic injury in these two groups without damaging the surrounding soft tissues.

## Methods

Five adult fresh cadaver knees were provided by the Department of Anatomy of Southern Medical University. X-ray examination was performed to confirm that there the patients had not received any surgery when they were alive and there were no apparent organic changes in the bones. There were 3 males and 2 females, with an average age of death of 35.4 years [(35.4 ± 5.7) years]. Their body weight was 65.2 kg [(65.2 ± 4.8) kg] and the average body height was 164.6 cm [(164.6 ± 4.5) cm] before death. The method to prepare the samples are shown in the following steps:

1. Preparation of specimens: Knee joints were harvested 20 cm up and down their edges. The articular capsule was cut open via the anteromedial incision to expose the knee joint. After the ACL tissue was resected, the incision was sutured, and the specimens were stored at − 40 °C for further experiments.

**Fig. 1:**
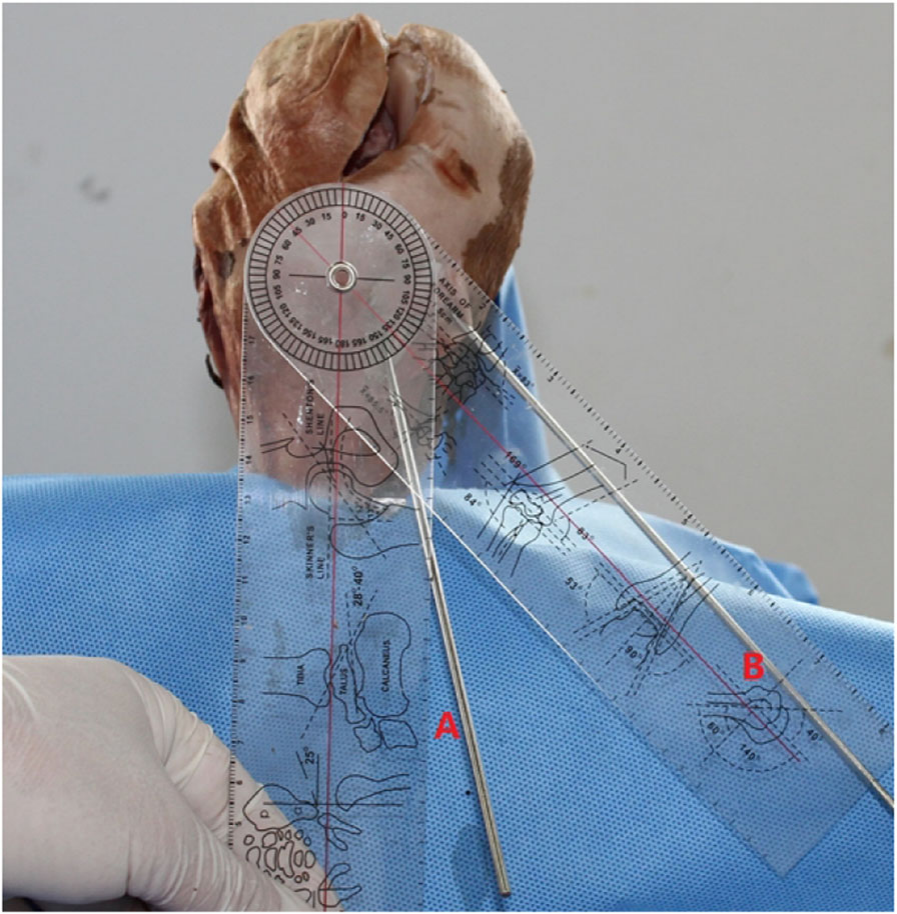
Two tibial tunnels are drilled in each specimen: A) at the 25° angle of coronal section; and B) at the 45° angle of coronal section)

Before the experiment, the specimens were placed at room temperature for 24 h. After the sutures of part of the anteromedial incision were divided, the tibial tunnel locator was placed at the midpoint of ACL under direct vision. The tibial guide was adjusted at 50° at the sagittal level, Two different tibial tunnel groups were established: in group A, the tunnel external aperture was placed at the 25° angle of coronal section, and in group B the tunnel external aperture was placed at the 45° angle of coronal section (Fig. 1). Tibial tunnels were obtained by using hollow drill and the Kirschner needle was inserted to measure the tunnel length, which was 43.8 ± 2.0 mm in group A and 45.2 ± 1.3 mm in group B.

**Fig. 2:**
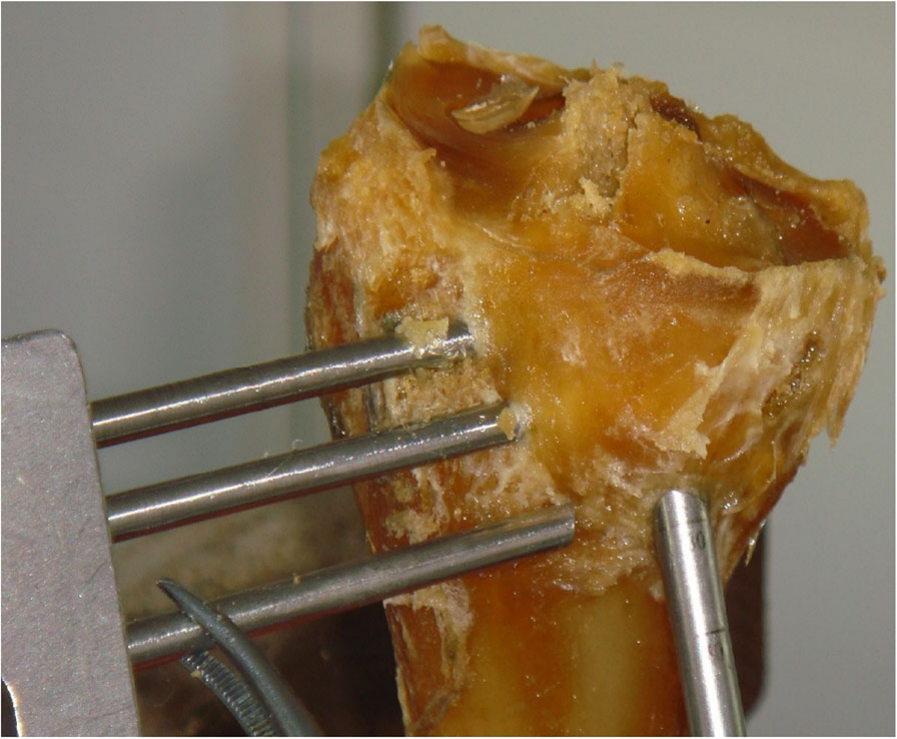
When the rotation angle of the guide is greater than 60°, less bone is penetrated through the sleeve of the cross pins and more is exposed

**Fig. 3:**
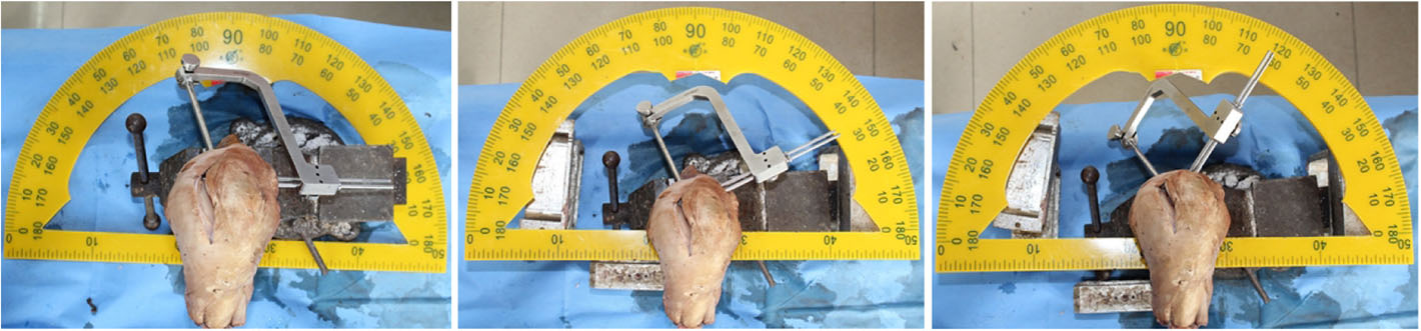
The placement angle of the cross pins was adjusted by rotating the angles of the Rigidfix guide

**Fig. 4:**
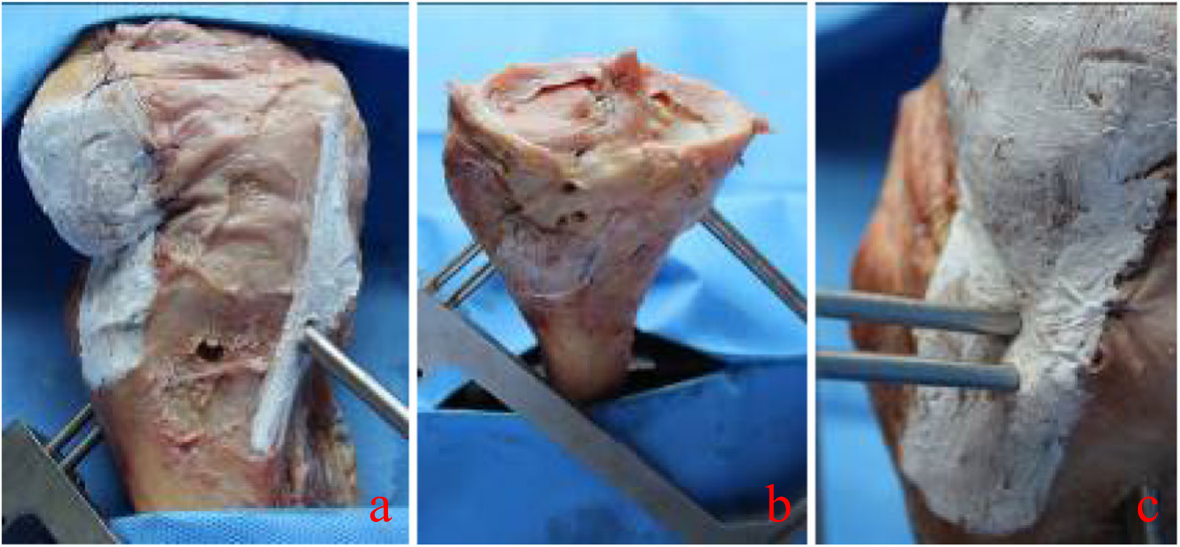
(**a**) The tibial tunnel injured the medial collateral ligament of knee joint; (**b**) the cross-pin breaks through the articular surface of tibial plateau; and (**c**) the cross-pin injures the patellar tendon

(2) Determining the angles of measurement: According to the results of our pre-experiment and previous experiments [11], we believe that when the rotation angle of the guide is greater than 60°, less bone is penetrated through the sleeve of the cross pins and more is exposed, even the furthest end of the sleeve cannot enter the bone. In this case, the sleeve cannot be fixed, and may easily shake or slide out. If the cross pin is inserted, it will inevitably cause the tail end of the cross nail to be exposed or slide out, as shown in Fig. 2. Therefore, we placed the guides in the two groups of tibial tunnels of A and B, rotated the angle of each group of guides at 0°, 30°, and 60° (Fig. 3), and then the cross pin tunnel was drilled. The incidences of iatrogenic injuries such as cartilage and ligament injuries at these three angles were observed.
(3) Definition of injury: The epidermis and subcutaneous adipose tissues were removed to observe the drilled tibial tunnel as well as to identify the relationships of the cross pins at tibial side with the adjacent ligaments, tendons, common peroneal nerve, popliteal nerve, and popliteal vein. The adjacent soft tissues were removed to expose the tibia to observe the cross pins had penetrated the posterior wall and medial wall of tibia or the tibial plateau. Observation of these 5 specimens showed that the cross-pin penetrated the posterior and medial walls of the tibia in none of the cases; both the tibial tunnel and the cross pins tunnel had a certain distance from the common peroneal nerve and fibular collateral ligament. However, it was also found that, when the tibial tunnel was placed at the excessively postersuperior site, it could injure the medial collateral ligament (MCL), and the cross-pins, when placed in the tunnel, had a risk of breaking through the tibial plateau and injuring the patellar tendon. Based on the above findings, we defined the MCL injury caused by tibial tunnel: the most medial border of the external aperture of tibial tunnel is in the traveling area of the tibial side of MCL of knee joint. As shown in Fig. 4 (a) and (b), tibial plateau cartilage (TPC) injury caused by cross pins: The cross-pin system (either proximal end or distal end) breaks through articular surfaces. Patellar tendon injury from Fig. 4(b) and (c) caused by cross pins: The cross-pin system (either proximal end or distal end) enters the traveling area of the patellar tendon. (Fig. 4)

(4) Procedures: The Rigidfix guide in groups A and B was placed at the lateral tibial plateau via different tibial tunnels. By rotating the guide inwards, the cross-pin was placed into the tunnel at three angles (0°, 30°, and 60°). The relationship of the tibial tunnel location with MCL and the relationships of the tunnel with the medial condyle of tibial plateau and patellar tendon were observed (Fig. 4). All operations were completed by a senior surgeon.

Then, statistical analysis was performed using SPSS 13.0 software. The incidences of MCL injury, TPC injury, and patellar tendon injury in two groups were compared by using chi square test, and a *P* value of < 0.05 was regarded as statistically significant.

## Results

**Fig. 5:**
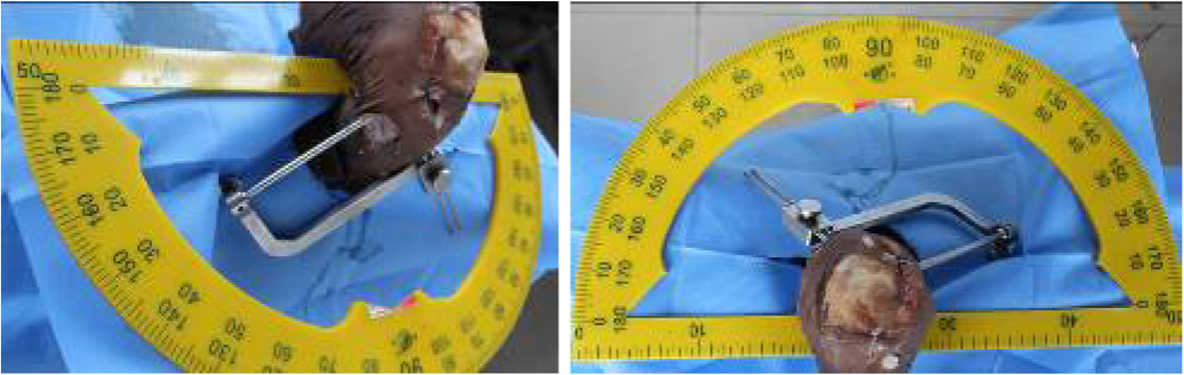
In group, the external aperture of the tunnel is located backwards and the starting rotation angle of the guide can not be 0°; thus, the guide was closely attached to the anterior crural region as the starting position to measure the starting angles at 35°- 45°(39.0 ± 6.5°)

Tibial tunnels were drilled in both groups to observe the relationship of the external aperture with MCL. No MCL injury was found in group A, and the incidence of MCL injury was up to 80% in group B (*P* = 0.048, Fisher exact probability method). When the Rigidfix system was applied for tibial fixation during ACL reconstruction, the Rigidfix guide passed through different tibial tunnels (A and B), and the guide was rotated at three angles (0°, 30°, and 60°). During the experiment, since the tunnel in group B was more prone to the back side, the rotation angle of the guide was restricted, and the starting position could not be 0°. Then, the guide was closely attached to the anterior crural region as the starting position (Fig. 5) to measure the starting angles at 35°- 45° (39.0 ± 6.5°). Notably, one specimen in group A had a starting rotation angle of only 30° due to thick subcutaneous fat. Thus, the number of measurements was different between group A and group B: 14 in group A and 10 in group B. The incidence of TPC injury was 21.4 and 70% in group A and group B, respectively, and the incidence of patellar tendon injury was 42.9 and 70%, respectively (Tables 1, 2 and 3). To identify the relationships of the tunnel external aperture in groups A and B with the surrounding structures, we also measured the distance between the external aperture and the medial margin of tibial tubercle, which was (19.2 ± 2.4) mm in group A and (38.3 ± 5.2) mm in group B.

**Table 1:** Statistical analysis of the incidence of MCL injury in tunnels between groups A and B

Group	Medial collateral ligament injury			*P*
	Yes	No	Incidence	
A	0	5	0.0%	0.048
B	4	1	80.0%	

Note: since *N* < 40 and T < 5, the Fisher exact probability method (two-sided) was adopted

**Table 2:** Statistical analysis of the TPC injury caused by Rigidfix cross-pin system through different tibial tunnels

Group	Angle	Penetration of the articular surface			*χ*^2^	*P*
		Yes	No	Incidence		
A	0	2	2	50.0%	5.662	0.017
	30	1	4	20.0%		
	60	0	5	0.0%		
	Total	3	11	21.4%		
B	35–45	5	0	100.0%		
	60	2	3	40%		
	Total	7	3	70%		
Total		11	14	41.7%

**Table 3:** Comparison of the incidence of patellar tendon injury in Rigidfix system

Group	Angle	Patellar tendon injury			*χ*^2^	*P*
		Yes	No	Incidence		
A	0	0	4	0.0%	0.120	0.729
	30	2	3	40.0%		
	60	4	1	80.0%		
	Subtotal	6	8	42.9%		
B	35–45	0	5	0.0%		
	60	3	2	60%		
	Subtotal	3	7	30%		
Total		9	15	37.5%		

The incidence of MCL injury was not significantly different between two groups (*χ*^2^ = 5.733, *P* = 0.057) (Table 1).

The incidence of TPC injury by cross pins through different tibial tunnels showed significant difference (*χ*^2^ = 5.662, *P* = 0.017) (Table 2).

Comparison of the patellar tendon injury between two groups showed no significant difference (*χ*^2^ = 0.120*, P* = 0.729) (Table 3). Although patellar tendon injury was found at 30° in two cases from group A, it was located at the edge of the lateral margin of patellar tendon. The average distance between the external aperture of the cross-pin tunnel and the lateral margin of patellar tendon was (5.54 ± 3.52) mm for proximal end and (3.00 ± 2.12) mm for distal end (Table 4).

**Table 4:** Distance between the external aperture of the cross-pin tunnel and the lateral margin of patellar tendon at 30° in group A

Specimen	Proximal pin (mm)	Distal pin (mm)
1	−1.5	−3
2	9.3	6
3	5	3
4	2.9	0
5	9	3
Absolute	5.54 ± 3.52	3.00 ± 2.12

Note: with the lateral margin of the patellar tendon as “zero” point, values within the traveling area of patellar tendon are negative and those outside the traveling area of patellar tendon are positive

## Discussion

ACL injury can cause knee instability, which can be accompanied by cartilage damage and ultimately knee dysfunction. Thus, ACL reconstruction is required to restore joint stability as much as possible and avoid any secondary injury of the joint. Proper graft selection is one of the key steps in ACL reconstruction. The use of hamstring tendons in ACL reconstruction can remarkably reduce the donor site complications such as anterior patellar pain and restricted extension and flexion; in addition, the four-strand tendons have similar elastic modulus with ACL and much higher strength and thus can effectively restore the stability of knee joint after reconstruction [14–17]. Tohyama et al. measured 16 adult fresh specimens and found the average lengths of the semitendinosus and the gracilis tendons were 235 ± 20 mm (mean ± SD) and 200 ± 17 mm, respectively, among which the shortest length of the gracilis muscle was 180 in one case, which could be 90 mm in length when applied for two-bundle four-strand reconstruction and thus met the requirement for reconstruction length [18]. Therefore, in recent years, hamstring tendons have increasingly been used, accounting for nearly half of ACL reconstruction [19]. According to our clinical experiences, the hamstring tendons can be easily ruptured during harvesting due to the presence of bundles and strands, resulting in short length. Therefore, other internal fixation methods such as internal interface screw fixation at the inner aperture of the tunnel are used instead. However, it is difficult to insert the interface screw into the inner aperture of the tunnel; in addition, the use of the interface screws can be accompanied with complications including cutting of the grafted tendon, micro-fracture of the tunnel wall, and offset of the reconstruction point [20]; also, it can increase the incidence of bone tunnel enlargement after operation [21]. All these conditions can reduce the effectiveness of surgery and, to a certain extent, restrict the application of the hamstring tendons. Therefore, a proper internal fixation method is critical for lowering the dependence on graft length during ACL reconstruction.

The commonly used devices for the internal fixation of grafts at femoral side include Endobutton steel miniplate, interface screw, and Rigidfix cross pins; at the tibial side, however, there are fewer options but can include interface screw and U-shaped nails. The fixing points of the interface screws and U-shaped nails are at the external aperture of the tunnels and far away from the physiological attachment point; thus, these two devices can not meet the requirements on physiological reconstruction and have higher requirements on graft length.

The Rigidfix cross-pin system, applied at the femoral side, has the following advantages: (a) since its fixing point is near the ACL anatomic attachment point, the Rigidfix cross-pin system can reduce the “rubber band effect” and “rain-wiper effect” and reduce the complications such as postoperative tunnel enlargement [1]; (b) the graft is exposed to the bone tunnel at 360°, which facilitates the healing of bone tendon; and (c) the fixation is firm and stable. Therefore, the Rigidfix cross-pin system has been widely recognized and applied in clinical settings [3, 4, 9, 22]. In Europe and the United States, the application of this system is increasing annually [23, 24]. Ahn et al. [25] used the Rigidfix system for the tibial side fixation during posterior cruciate ligament (PCL) reconstruction and believed that this procedure could reduce the dependence on the graft length and achieve firm and stable fixation. Then, can the Rigidfix system be applied to the tibial side fixation during ACL reconstruction? Antonogiannakis has tried in this respect [9]. He applied Rigidfix system for both tibial side fixation and femoral side fixation and concluded that a 8–9 cm-long quadriceps tendon could meet the requirement on reconstruction. Liu et al. [26] applied the Rigidfix system for femoral side fixation in tibial side fixation. They argued that the tip of the guide should be placed at subchondral bone, but without specific requirements on the guide angle. After following 32 patients, they believed that this method can make up for the shortcomings of other fixation methods (e.g. interface screws and bolt piles), allow the complete contact between tendon and bone tunnel, and facilitate the healing of bone and tendon. Postoperative MRI examination revealed that the fixation point was close to the joint line and no bone enlargement was found.

According to our experiences, internal fixation at either femoral or tibial side is near attachment point of the articular surface. At the femoral side, the cross pins can penetrate the articular surface no more than 20 mm. At the tibial side, if the guide tip of the Rigidfix system is placed beneath the articular cartilage surface, the distance between the fixation point and the articular surface will be no larger than 17 mm, and the tendon thickness (about 3 mm) should be added at both sides. Therefore, the required intratunnel length of the tendon is only 43 mm; after the intraarticular length (27 mm) [27] of the tendon is added, the required length of the grafted tendon is only 70 mm, which can meet the requirement of ACL reconstruction and dramatically lower the dependence on graft length during this procedure. Traditionally, the femoral side was fixed with the Rigidfix system, during which the graft length is at least 28 mm in femoral tunnel and 40–50 mm in tibial tunnel; the total length of the graft should be at least 90 mm–95 mm.

In our opinion, the Rigidfix system, when applied at the tibial side during ACL reconstruction, also is firm and stable and can achieve 360° to tendon/bone. When this system is used at both femoral and tibial sides, the fixation point is close to the ACL anatomic attachment point, which meets the requirements of physiological reconstruction and meanwhile decreases the requirement on graft length. Some authors have succeeded in the clinical application of the Rigidfix system [10, 27]. However, when the Rigidfix cross pins are used to fix the grafted tendon, the pins must pass across the center of the graft to achieve the suspension fixation of the tendon. The length of the pins inserted is 3.5 cm, which must be all located inside the bone substance, otherwise these pins may protrude into subcutaneous tissues or joints. Then, the question is: will the similar phenomenon occur if the Rigidfix system is applied to fix the tendon at the tibial side during ACL reconstruction? In one of our previous studies, we performed anatomic study on 11 antiseptic specimens and found that, when the guide of the Rigidfix system was placed 0.5 cm beneath the articular surface, it could lower the risk of penetrating tibial plateau, along with lower probability of penetrating the medial tibial cortex. Therefore, we believe that placing the Rigidfix cross pins 0.5 cm beneath the articular surface is more feasible [11]. In recent years, however, with an attempt to drill an ideal femoral tunnel through the tibial tunnel, the location of the tibial tunnel was moved upwards, with an angle of 40° - 45° on coronal plane [12]. Based on the previous studies, in our current study we further explore the potential effect of different tibial tunnels on the application of the Rigidfix system at the tibial side and any difference in the incidences of iatrogenic injuries.

In group A, the longitudinal angle of tibia was set at 25° as the external aperture of the tibial tunnel. After the tunnel was drilled, the distance between tibial tunnel and medial margin of tibial tubercle was measured (mean: 19.2 ± 2.4 mm). Based on our previous experimental experiences [11], we set the rotation angles of the Rigidfix cross-pin guide at 0°, 30°, and 60°. However, it was found that the skin and subcutaneous fat were preserved in the fresh specimens; compared with the antiseptic specimens, the fresh specimens had more water. The Crus anterior restricted the rotation of the guide. In group A, the guide could not be rotated to 0° in one case; in group B, the backward movement of the tibial tunnel caused the backward placement of the guide, which further restricted its rotation angle. As a result, the starting angle could only begin from35°- 45° (39.0° ± 6.5°).

In group B, the posterior-superior displacement of the external aperture of the tunnel was associated with higher risk of MCL injury when compared with group A (*P* = 0.048). (Table 1) Also as seen in our experiment, larger slope angle led to larger angle between cross-pin tunnel and tibial plateau, which could further increase the incidence of the injury of the articular cartilage of tibial plateau. In group A, along with the decrease in the angle of the tibial tunnel, the incidence of the injury of articular surface of tibial plateau also significantly decreased (*χ*^2^ = 5.662, *P* = 0.017, compared with group B). (Table 2) Although the incidence of patellar tendon injury showed no significant difference between groups A and B (*χ*^2^ = 0.120, *P* = 0.729), it gradually increased along with the internal rotation (0°-60°) of the guide in the tibial tunnel. When the rotation angle reached 60°, the incidence of patellar tendon injury reached 80% in group A and 70% in group B. In group A, two cases had patellar tendon injury when the rotation angle was 30°, and the injury was located at the lateral margin of patellar tendon in one case and 3 mm within the lateral margin of the patellar tendon in the other case. (Table 3) Also in group A, when the rotation angle was 30°, the distance between the site of cross-pin placement and the lateral margin of patellar tendon was (5.54 ± 3.52) mm for proximal pins and (3.00 ± 2.12) mm for distal pins (Table 4). Meanwhile, the incidence of the injury of tibial plateau cartilage in group A was 20% (1/5), which was relatively low.

It is therefore concluded that when the Rigidfix cross pin system is used for tibial fixation during ACL reconstruction, the tibial tunnel should not be placed at the excessively postersuperior site, so as to lower the incidences of MCL and TPC injuries. The guide is placed 5 mm below the articular surface. When the guide rotates inwards, the incidence of patellar tendon injury gradually increases along with the increase of the rotation angle. We observed the relationship between cross-pin placement role and lateral margin of patellar tendon when the rotation angle was 30° and found that the incidence of TPC injury was relatively low when the cross-pins were placed near the lateral margin of patellar tendon [(5.54 ± 3.52) mm for proximal pins and (3.00 ± 2.12) mm for distal pins]. (Table 4) Therefore, 30° is a feasible angle for the placement of cross-pins.

Although an effect of the cross-pin placement angle on the iatrogenic injuries was observed in this study, a statistical analysis was not possible due to the small sample size of our study. When the Rigidfix cross-pin system is applied for fixing the tibial side during ACL reconstruction, MCL injury, TPC injury, and patellar tendon injury may occur. To avoid the MCL and TPC injuries, the tibial tunnels should not be placed at the excessively postersuperior site. A longitudinal angle of tibia set at 25° is more feasible (about 20 mm away from medial margin of tibial tubercle). With the increase of the internal rotation angle of the Rigidfix guide, the incidence of TPC injury will drop, whereas that of patellar tendon injury gradually increases. Therefore, the external aperture of tibial tunnel should be about 20 mm away from the medial margin of tibial tubercle. The cross-pin placement tunnel can be drilled when the Rigidfix guide is rotated to 30° (or 3 - 5 mm away from the external margin of patellar tendon), which can reduce the incidences of TPC injury and patellar tendon injury.

### Limitations

Although we observed the trend of the impact angle of the cross pins on the iatrogenic injury, the effect of different placement angles of the cross pins on the iatrogenic injury could not be carried out using statistical analysis due to the small number of specimens in this study.

## Conclusion

We studied the safety of the application of the Rigidfix cross-pin system via different tibial tunnels in the tibial fixation during ACL reconstruction. It is fond that when the Rigidfix cross-pin system is used for ACL reconstruction at the tibial fixation site, the external aperture of tibial tunnel should not be placed at the excessively posterosuperior site. Then the it can reduce the possibility of the MCL and TPC injuries.

## Data Availability

The datasets used and analyzed during the current study are available from the corresponding author on reasonable request.

## Abbreviations

ACL: Anterior cruciate ligament
TPC: Tibial plateau cartilage
MCL: Medial collateral ligament
